# CELL NON-AUTONOMOUS MECHANISMS OF AGING

**DOI:** 10.1093/geroni/igz038.2675

**Published:** 2019-11-08

**Authors:** Scott Leiser

**Affiliations:** University of Michigan, Ann Arbor, Michigan, United States

## Abstract

It is now recognized that biological aging can be affected both positively and negatively by intercellular communication. This symposium will focus on recent discoveries related to cell non-autonomous mechanisms of aging.

